# The New Visual Erection Hardness Score is the Topic Highligheted in this Issue of International Brazilian Journal of Urology

**DOI:** 10.1590/S1677-5538.IBJU.2025.02.01

**Published:** 2025-02-26

**Authors:** Luciano A. Favorito

**Affiliations:** 1 Universidade do Estado do Rio de Janeiro Unidade de Pesquisa Urogenital Rio de Janeiro RJ Brasil Unidade de Pesquisa Urogenital - Universidade do Estado do Rio de Janeiro - Uerj, Rio de Janeiro, RJ, Brasil; 2 Hospital Federal da Lagoa Serviço de Urologia Rio de Janeiro RJ Brasil Serviço de Urologia, Hospital Federal da Lagoa, Rio de Janeiro, RJ, Brasil

The March-April 2024 number of Int Braz J Urol is the thirty-thirdunder my supervision. In this number the Int Braz J Urol presents original contributions with a lot of interesting papers in different fields: Bladder Cancer, Bladder dysfunction in Children, Renal cell Carcinoma, Intra-renal surgery, Cistoscopy, Prostate Biopsy, Gender affirming surgery, urogenital tuberculosis and erectile dysfuncion. The papers came from many different countries such as Brazil, USA, Italy, Turkey, Egypt, Serbia and China, and as usual the editor's comment highlights some of them. The editor in chief would like to highlight some papers in this number, specially the papers about the new visual erection hardness score:

Dr. Figueiredo and collegues from Brazil, presented in e20240590 (1) a nice review about urogenital tuberculosis (UGT) and concluded that the diagnosis of UGT depends on a high degree of suspicion based on non-specific symptoms and radiological findings. Urinary bacteriological tests have low sensitivity, but even in the absence of diagnostic confirmation, treatment can be carried out through a combination of drugs for a period of six months. In the presence of ureteral stenosis or contracted bladder, complex but well stablished reconstruction procedures are necessary.

Dr. Lopes and collegues from Brazil performed in e20240490 (2) a nice systematic review about pelvic lymph node dissection before versus after radical cystectomy and concluded that the timing of the lymphadenectomy was not associated with a significant reduction in total operative time, pelvic lymph node dissection (PLND) time, number of LN dissected, and estimated blood loss.

Dr. Alam from the group of Dr. Arthur Burnett from USA, presented in e20240332 (3) presented a important study about important systematic review about the penile prosthesis (PP) placement with concomitant non-reconstructive urologic procedures and concluded that patients undergoing PP implantation with a concomitant non- reconstructive urologic procedure, had no increased risk of complications or device infections when compared to patients undergoing first-time PP placement only. While further investigation is needed, our findings challenge the traditional dogma that secondary urologic procedures should be avoided at the time of PP implantation.

Dr. Mansour and collegues from Egypt and USA showing in e20244425 (4) a nice, randomized trial about the efficacy and safety of mirabegron compared to solifenacin in treatment of non-neurogenic overactive bladder in children and concluded that Mirabegron is more effective with fewer treatment-related adverse effects compared to solifenacin in children with OAB refractory to behavioral therapy and other anticholinergic medications. Mirabegron treatment improves daytime symptoms and nocturnal enuresis with less risk of constipation. It may be considered as a first-line pharmacotherapy for select patients with non-neurogenic OAB.

The group of Dr. Ho from Serbia and USA performed in e20240427 (5) an interesting study about testicular implant complications after Transmasculine Gender Affirming Surgery and concluded that complications after testicular implants in transgender men are not uncommon events. The present paper suggests that implant size is not a significant predictor of complications requiring prosthetic removal.

Dr. Carbonara and collegues from Italy and USA performed in e20240565 (6) a nice study about Percutaneous cryotherapy (CRYO) and radiofrequency ablation (RFA) of renal masses: multicenter comparative analysis with minimum 3-year follow-up and concluded that CRYO and RFA are both valid minimally invasive options for the treatment of small renal tumors. They are particularly suitable for patients who are not good surgical candidate as they offer very low risk of major procedure-related complications. For the right indication, they both offer favorable mid to long term oncologic outcomes.

Dr. Zhou and collegues from China performed in e20240630 (7) a very nice study about Biplanar or Monoplanar Prostate Biopsy: Should Transrectal and Transperineal Approaches Be Combined for Prostate Cancer Detection ? and concluded that biplanar stereotactic biopsy was superior to monoplanar biopsy in detecting anterior csPCa. Both methods demonstrated no significant differences in overall PCa detection rates and safety.

Dr. Westin and Collegues from Urogenital Research Unit – Brazil performed in e20249923 (8) an interesting study about bladder mucosa harvested with holmium laser for treatment of urethral strictures: does the graft have its tissue integrity preserved? and concluded that the graft harvested from the bladder uroepithelium using Ho-YAG has its histological integrity preserved, which makes this technique a viable option for reconstructive surgery. However, more studies are needed to establish its long- term efficacy and safety of this new technique.

Dr. Schuh and collegues performed in e20249927 (9) the cover paper of this edition: a nice study about shock wave therapy (LI-ESWT) in the treatment of erection dysfunction: how to define clinical outcomes? a comparison between penile doppler ultrasound and a new visual erection hardness score (v-ehs) during a blinded, sham-controlled trial and concluded that LI-ESWT has proven effective in the treatment of moderate vasculogenic erectile dysfunction, with optimal results at 6 months. The new V-EHS offers a simple, reliable and reproducible assessment of erectile function.

The Editor-in-chief expects everyone to enjoy reading.
